# Differential game in closed-loop supply chain of innovative products with double regrets of consumers

**DOI:** 10.1371/journal.pone.0347690

**Published:** 2026-05-22

**Authors:** Qing Ma, Yao Peng, Zhenwei Liu

**Affiliations:** 1 Department of Business Administration, Moutai Institute, Renhuai, China; 2 School of Economics and Management, University of Electronic Science and Technology of China, Chengdu, China; Harbin Institute of Technology, CHINA

## Abstract

In this paper, we consider a three-level dynamic closed-loop supply chain differential game model led by manufacturers and recycled by third-party recyclers in order to study the impact on the dynamic closed-loop supply chain of innovative products based on simultaneous occurrence of consumer purchase regret and replacement regret. Then, we obtain the optimal path for wholesale price, retail price and return effort by solving the differential game model. Specifically, we explore the influence of the coupling effect of consumer purchase regret and replacement regret on the supply chain through theoretical analysis and numerical simulation. The results show that consumer purchase regret leads to lower sales in the supply chain, which in turn causes a decrease in long-term profits of manufacturers and retailers, while consumer replacement regret will attract more consumers to increase sales, thereby increasing the long-term profits for manufacturers and retailers accordingly. However, consumer purchase regret does not affect the profit of recyclers. In addition, both types of consumer post-purchase regret prolong the product length of time in the market, and consumer replacement regret has a more significant effect on the length of time of product in the market. Meanwhile, lower consumer purchase regret and higher replacement regret can improve the performance of all members of the entire dynamic supply chain. However, these factors do not influence the recyclers.

## 1. Introduction

The contradiction among social development, resources and environment is growing increasingly acute. How to achieve green, low-carbon and circular development, improve resource utilisation efficiency and reduce the level of environmental pollution are a common concerns of all sectors of the society. The closed-loop supply chain management model, which makes rational use of recycling and remanufacturing of waste products, is undoubtedly a good prescription for solving the above-mentioned “chronic problems”. With increasing emphasis on sustainable development and the environment protection, remanufacturing has become an important strategy for enterprises to improve their core competitiveness. The market for remanufactured products is growing significantly [1]. It is estimated that the global value of remanufactured goods exceeds $100 billion annually, with the consumer market accounting for $10 billion in sales [2]. Over the past few decades, rising interest in environmentally friendly practices and growing customer awareness of remanufactured products have encouraged companies to adopt closed-loop supply chains management [3].

It is noteworthy that the realization of environmental and economic benefits in closed-loop supply chains relies not only on advancements in recycling technologies and the optimization of remanufacturing processes, but also more crucially on deep engagement in the level of consumer behavior. Consumers’ post-purchase psychological responses to innovative products (such as satisfaction and regret) can significantly influence product lifecycle management through word-of-mouth effects. Particularly when consumers experience post-purchase regret due to unmet expectations or replacement regret towards competing products, their subsequent behaviors may trigger structural shifts in market demand, which potentially inhibiting the sustained diffusion of innovative products or affecting potential consumers’ purchase intentions through negative evaluations of these competing products. This dynamic interaction between psychological behaviors on the consumption side and decision-making at the production side has evolved into an intangible yet critical “hidden nexus” for maintaining the long-term stability of closed-loop supply chains.

Previous literature has predominantly studied closed-loop supply chains from a static perspective. As research progresses, it has been recognised that the relationships between decision-makers in these systems constitute a long-term dynamic cooperative game process. In addition, previous studies seldom consider the mindset of consumers after purchasing a product. As a matter of fact, different consumers will have different psychological reactions after purchasing a product. Some of them may experience regret, and different regret behaviours will directly affect the later sales of the products and the long-term benefits of each decision maker in closed-loop supply chains. Therefore, integrating consumers’ post-purchase regret psychology into closed-loop supply chain models holds significant theoretical and practical value.

This paper examines the impact of two types of post-purchase regret on supply chain performance. The first type occurs when consumers purchase an innovative product and subsequently realise it fails to meet their expectations. For example, they may struggle to utilise many of the product’s innovative features, feeling they have overspent on non-essential functionalities, resulting in regret. Alternatively, after purchasing the product, consumers might discover that certain features underperform compared to advertisements, perceiving the purchase as wasteful due to unmet expectations. This phenomenon is termed purchase regret in this study. The second type of post-purchase regret arises when consumers buy competing products and later find these alternatives inferior in functionality, leading to regret. These regretful consumers then become potential buyers of the innovative product. Such regret is defined as replacement regret in this paper.

Based on the aforementioned context, this paper examines the impact of post-purchase regret on each member of the supply chain and their decisions, and addresses the following questions: 1.How does the phenomenon of consumers’ post-purchase regret influence the pricing policy of supply chain members? 2.What is the impact of the phenomenon of consumers’ post-purchase regret on the market diffusion and sales volume of the innovative product? 3.Can the phenomenon of consumers’ post-purchase regret have a beneficial impact on enterprises? To address the above questions, we constructed a closed-loop supply chain comprising a manufacturer, a retailer, and a third-party recycler. In this structure, the manufacturer acts as the leader of the supply chain, while the retailer and recycler serve as followers. The manufacturer distributes products through the retailer and relies on the third-party recycler for product recovery. Our analysis reveals the following novel insights.

First, the manufacturer’s wholesale pricing strategy and the recycler’s recycling effort strategy remain unaffected by both types of consumer post-purchase regret behaviors. However, these behaviors influence the retailer’s retail pricing strategy. When consumer purchase regret occurs, the retailer raises the retail price to offset profit losses caused by consumer regret. Conversely, when consumer replacement regret arises, the retailer lowers the retail price to attract more consumers, thereby gaining greater profits.

Second, the consumer purchase regret will lead to a decline in the sales of the innovative product, because consumer purchase regret behaviour affects the spread of the innovative product among consumers and slows down the diffusion of the product in the market, which leads to a decline in the final total sales of the product, and further leads to a decline in the manufacturer‘s long-term profit; the retailer can only make up for some of the lost profit caused by consumer purchase regret by increasing the retail price, but it cannot recoup all the losses caused by the consumer purchase regret, which leads to a decline in its long-term profit.

Finally, the consumer replacement regret will lead to an increase in the sales of the innovative product due to the dissatisfaction of the consumers of the competing products, thus indicating that the innovative product is better than the competing product, which will influence the potential consumers of the better innovative product to buy the innovative product, resulting in higher total final sales of the product, and further higher long-term profits for the manufacturer and retailer.

### 1.1. Literature review

This work is related to two streams of literature: closed-loop supply chains and consumer regret. We first provide an overview for each literature stream and then summarize our contribution.

Existing closed-loop supply chains studies are mainly from two perspectives, static and dynamic. Static closed-loop supply chains studies consider the optimal decisions of supply chain members in the short term, and believe that the optimal decisions are fixed in the short term without changing over time. Savaskan et al. [4] firstly proposed a static model of closed-loop supply chains and considered recycling by a manufacturer, a retailer and a third-party recycler respectively. Ma et al. [5] studied static closed-loop supply chains consisting of a manufacturer, a retailer and two recyclers and considered the alliance structure of different supply chain members. Zhang and Chen [6] considered government subsidies and scale effects in a closed-loop supply chain with third – party recycling to investigate the optimal decision-making and coordination issues of members. Zhu and Li [7] studied a two-sided platform consisting of manufacturers and recyclers and investigated the pricing strategies of closed-loop supply chains under both centralized and decentralized decision-making. With the development of the Internet and the rise of e-commerce, many manufacturers have opened direct sales channels and adopted dual-channel sales strategies. This trend has spurred research on dual-channel closed-loop supply chains which have garnered significant scholarly attention [8–10].

In contrast, Dynamic Closed-loop Supply Chains focuses on the optimal decisions of the members of the supply chain in the long run, believing that their optimal decisions are not fixed but change over time and there exists an optimal decision path [11]. Lee et al. [12] earlier studied the dynamic closed-loop supply chains model and considered the recycling situation of retailers. Giovanni et al. [13] studied the dynamic closed-loop supply chains where the manufacturer and the retailer simultaneously invest in product recycling. Ma and Hu [14] considered the second-level closed-loop supply chains differential games model under three behavioural models: manufacturer altruism, retailer altruism, and both altruism. Cao et al. [15] constructed a closed-loop supply chains differential games model for retailer recycling from the perspectives of manufacturer’s concern for retailer’s equity concerns and manufacturer‘s lack of concern. Wang et al. [16] studied the retailer-led, manufacturer-recycling closed-loop supply chains under the governmental reward and punishment mechanism and considered the stochastic evolution of the recycling rate. Liu et al. [17] considered the impact of the dual reference effect on the closed-loop supply chain and adopted a two-way cost-sharing contract for coordination. Huang et al. [18] investigate reverse channel selection within a closed-loop supply chain, and analyzed the impact of different collection channels on price, return rate, demand, and member profits.

However, most of the above dynamic supply chain research assumes that the product market is relatively mature, and uses the classical demand model or goodwill model to portray changes in demand. However, in real life, after a category of innovative products go on the market (e.g., each new model of Apple mobile phone, Tesla car), there exists the behaviour of consumers who have purchased that new model of product, which will have an impact on those who have not purchased that model of product. Based on this, the diffusion of innovation model was proposed by Bass [19] in 1969 to describe this behaviour. And Robinson and Lakhani [20] further considered the effect of the price factor on the basis of Bass’s model. Luigi and Andrea [21] applied Bass’s model, which takes into account the price factor, to the game model between a monopolistic innovative products firm and the government. Quan [22] introduced the Bass model into an open-loop supply chain and constructed a differential games model in which the manufacturer leads and the retailer follows. Therefore our work contributes to this stream of literature in several aspects. First, by incorporating the market diffusion of innovative products into closed-loop supply chain modeling, we extend existing frameworks that primarily focus on static environments or conventional products. Second, we investigate the impact of consumer regret phenomena (both purchase regret and replacement regret) on the decision-making of closed-loop supply chain members, revealing nuanced behavioral interactions previously unaddressed in the literature.

Our work also contributes to the literature on consumer regret. Consumer regret has established itself as a critical psychological determinant in consumer behavior research, consequently attracting intensified scholarly scrutiny [23]. Syam et al. [24] constructed a game model to study the relationship between consumers’ anticipated regrets and product standardisation; Nasiry and Popescu [25] used a linearised structure to describe the degree of “action regret” and “inaction regret”, and further analysed the impact of these two types of anticipated regrets on consumer purchasing decisions. Kuang and Fu [26] studied the impact of consumers’ anticipated regrets on the pricing, inventory and return decisions of online retailers in a random demand environment, and explored the return policies of online retailers. Zhang et al. [27] researched the impact of consumer regret expectations on the supply chain when prices are stimulated by discounts. Chen and Guan [28] considered the impact of strategic consumers’ anticipated regret behaviors on their purchase decisions and the operational decisions of online retailers, and studied the market coverage strategies and return policy of online retailers. Most research on regret focuses on consumers’ anticipated regrets [29], whereas post-purchase regret remains understudied. However, in consumer markets, post-purchase regret exerts a more substantial impact on product diffusion. Consequently, diverging from prior studies, our work examines how two types of post-purchase regret (purchase regret and replacement regret) influence decision-making and profitability across supply chain members.

The literature review above indicates three main limitations in current research: First, studies on dynamic closed-loop supply chains fail to consider the diffusion patterns of innovative products; Second, the influence mechanisms of consumers’ post-purchase regret behaviors have not been systematically revealed; Third, the integration of innovation diffusion models with dynamic game theory in closed-loop supply chains urgently needs to be explored. Table 1 presents a comparison between this work and previous literature. This paper effectively addresses these research gaps by constructing a differential game framework incorporating the Bass model and systematically analyzing the impacts of two types of post-purchase regret behaviors.

**Table 1:** A comparison table of Model assumptions among the present article and related previous articles.

Article	Dynamic CLSC	Bass Model	Post-purchase Regret
Savaskan et al. (2004)	×	×	×
Ma et al. (2016)	×	×	×
Zhang and Chen (2021)	×	×	×
Zhu and Li (2021)	×	×	×
Lee et al. (2011)	✓	×	×
Giovanni et al. (2016)	✓	×	×
Ma and Hu (2020)	✓	×	×
Cao et al. (2020)	✓	×	×
Wang et al. (2022)	✓	×	×
Liu et al. (2024)	✓	×	×
Huang et al. (2024)	✓	×	×
Liu et al. (2023)	✓	✓	×
This Work	✓	✓	✓

## 2. Basic theory

### 2.1. Optimal control theory [30–32]

The development of optimal control theory began with the proposal of the concept of “optimization”. During and after World War II, due to the needs of war and military defense, the technology of automatic control systems with the main goal of improving the hit rate of cannon firing was gradually perfected. However, with the development of society, simple feedback control had been difficult to meet the requirements of engineering practice, and traditional system design methods were unable to achieve the increasingly higher performance indicators. Under such circumstances, through a large number of studies, scientists put forward the concept of optimization in the early 1950s and attempted to exert optimal control on controlled objects. Around 1960, due to the introduction of a series of new research methods and mathematical achievements into control theory and the derivation of the sufficient conditions that optimal control must satisfy, the application of optimal control gradually became popular.

The problems studied by optimal control theory can be summarized as follows: For a controlled dynamic system or motion process, find an optimal control scheme from a class of allowed control schemes, so that when the motion of the system transfers from an initial state to a specified target state, the value of its performance index is optimal. Such problems widely exist in the technical field or social issues. In order to solve the optimal control problem, it is necessary to establish the motion equation describing the controlled motion process, give the allowable value range of the control variable, specify the initial state and target state of the motion process, and stipulate a performance index for evaluating the quality of the motion process.

The most basic optimal control problem is described as follows:


$$\begin{array}{c} maxV=\int_{\text{0}}^{T}F(t,y,u)dt \end{array}$$

$$\begin{array}{c} s.t. \end{array}$$

$$\begin{array}{c} \dot{y}(t)=f(t,y,u) \end{array}$$

$$\begin{array}{c} y(\text{0})=A,y(T)free(A,Tgiven) \end{array}$$

$$\begin{array}{c} u(T)\in UForallt\in [\text{0},T] \end{array}$$ (1)

Where t is the time variable, y is the state variable, u is the control variable, and λ(t) the covariate, the lagrangian coefficient. Accordingly, the Hamilton function can be constructed. The Hamilton function is defined as follows:


$$\begin{array}{c} H\left(t,y,u,\lambda \right)\equiv F\left(t,y,u\right)+\lambda \left(t\right)f\left(t,y,u\right) \end{array}$$ (2)

The most important aspect of solving the optimal control problem is the first order condition, also known as the maximum value principle. After defining the Hamiltonian function, the maximum principle conditions are as follows:


$$\begin{array}{c} \underset{u}{Max}H(t,y,u,\lambda )\text{For all }t\in [\text{0},T] \end{array}$$

$$\begin{array}{c} \dot{y}(t)=\frac{\partial H}{\partial \lambda }(\text{The equation of motion for }y) \end{array}$$

$$\begin{array}{c} \dot{\lambda }(T)=-\frac{\partial H}{\partial y}(\text{The equation of motion for }\lambda ) \end{array}$$

$$\begin{array}{c} \lambda (T)=\text{0}\left(\text{Transversality condition}\right) \end{array}$$ (3)

The symbol $\underset{u}{max}H$ indicates that the Hamiltonian function is maximised and this equivalence condition is expressed as:


$$\begin{array}{c} H\left(t,y,u^{*},\lambda \right)\geq H\left(t,y,u,\lambda \right)\left|\right||Forall\text{\textbackslash{} }t\in \left[0,T\right] \end{array}$$ (4)

$u^{*}$ is the optimal control quantity. If $\underset{u}{Max}\text{\textbackslash{}hspace\{0.17em}\}H\left(t,y,u,\lambda \right)$ is differentiable about u, it can be expressed by the first order condition $\frac{\partial H}{\partial u}=0$. The optimal decision variables, state variables, and covariates of the optimal control problem can be solved by the maximum value principle condition.

### 2.2. Bass model

The core concept of the Bass model is that the initial group purchasing a certain innovative durable product is considered the innovator group, whose purchasing decisions are independent of other members of the social system. Subsequent purchasers of the durable product form the imitator group, whose purchasing time of innovative products is influenced by the innovator group, and this influence increases as the number of purchasers grows. In the context of achieving the “dual carbon goals”, customers who are the first to use remanufactured products can be seen as the innovator group, while the other group can be seen as the imitator group. This theory is derived from the virus propagation model, where innovative products are equivalent to pathogens (viruses), and the attractiveness of innovative products to customers is equivalent to the virulence of a virus. The innovator group after a certain point in time consists of two parts: the number of people instantly infected by Innovative Products at that time and the cumulative number of infected people before that time, which is equivalent to infected individuals in the virus propagation model, while the imitator group corresponds to susceptible individuals. The mathematical foundation of this theory comes from the reliability function model in reliability theory. Equating the total market demand to the “total number of experiments” in the reliability function, the number of products sold at time t is equivalent to the “cumulative number of failed products” in the reliability function, and the number of products unsold at time t is equivalent to the “number of products still intact” in the reliability function. The market share of Innovative Products at time t is equivalent to the probability distribution function F(⬝) in the reliability function. Therefore, using the Bass model can effectively depict the evolution process of closed-loop supply chains dynamic differential games of new durable products.

Assuming that the law of innovative products after entering the market obeys the Bass model, that is, there is a process of diffusion of innovative products among consumers, and the total number of potential consumers in the market is N. Assuming that each consumer will only purchase the innovative product once, and call the consumers who have purchased the product innovators and those who have not purchased the product are called Imitators; thus the proportion of Innovators at time t is x(t) and satisfies $\dot{x}\left(t\right)=\left(j+kx\left(t\right)\right)\left(1-x\left(t\right)\right)$ (Reference [12]), j,k>0,x(0)=0,$\underset{t\to \infty }{\text{lim}}x(t)=\text{1}$. Where j is the consumer preference coefficient, for the time t after the launch of the new product, the proportion of the new product can be sold without any sales efforts, equivalent to the proportion of the population that is instantly infected in the viral transmission model at the time t; k is the imitation coefficient, which indicates the influence coefficient of the innovator on the imitator after the influence of sales efforts (equivalent to the number of basic regeneration in the viral transmission model); we can get the time t of the innovative products‘ demand $D\left(t\right)=N\dot{x}\left(t\right)$. According to the reference [13], consider that the demand elasticity ε is negatively correlated with the price p, that is $\varepsilon =\frac{p}{D}\frac{dD}{dp}\propto -p$,obtain $\frac{\text{1}}{dD}\propto -dp$, further $D\propto e^{-ap}$, that is, the quantity demanded is proportional to $e^{-ap}$, and because of the quantity demanded D(t)=N\stackrel·x(t), it can therefore be assumed that $\dot{x}\left(t\right)=e^{-ap\left(t\right)}\left(j+kx\left(t\right)\right)\left(1-x\left(t\right)\right)$, where a is the impact coefficient of price on the switch from innovators to imitators, that is, the consumer‘s price sensitivity coefficient.

## 3. Model assumption

**Fig 1:**
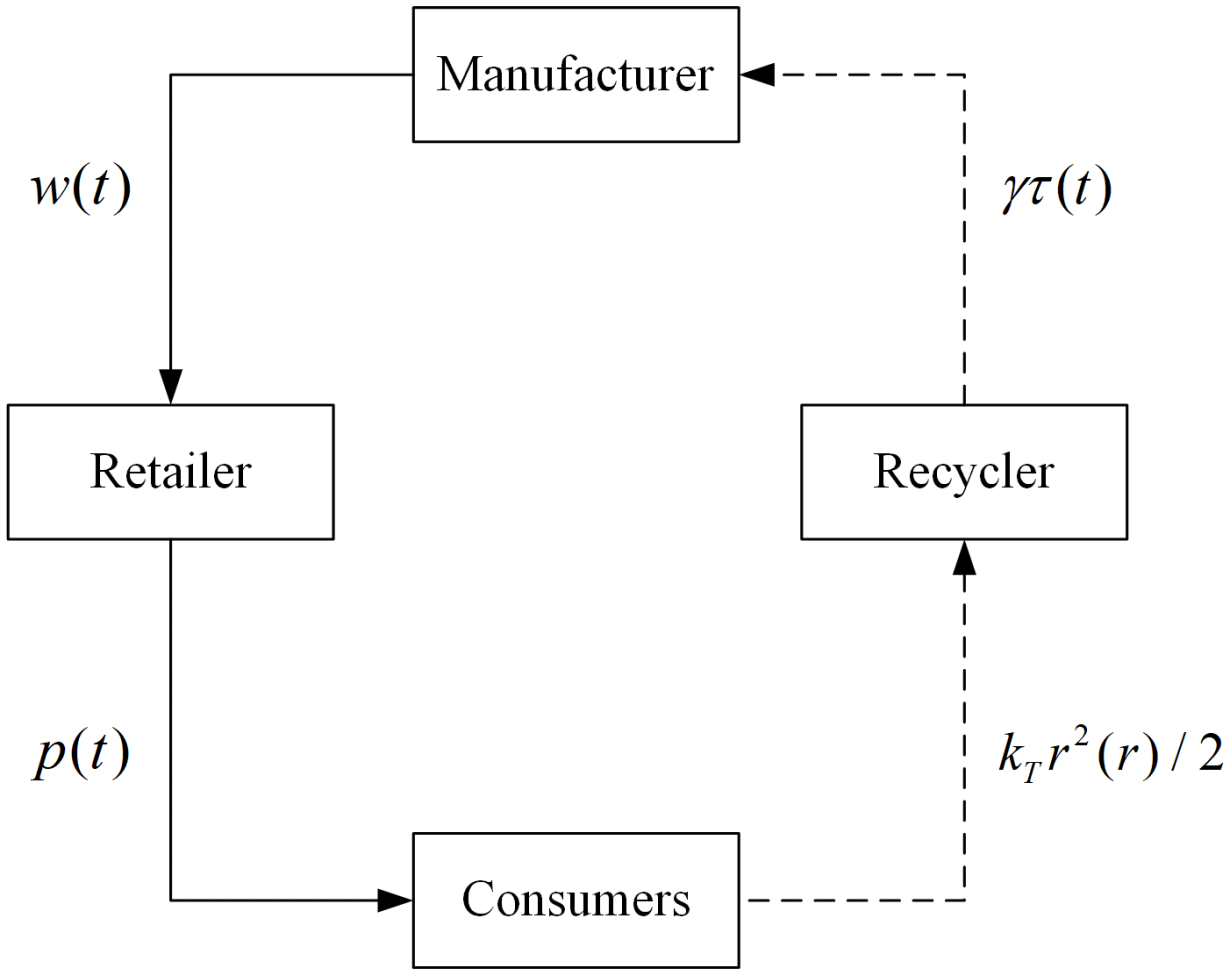
Supply chain structure diagram.

This paper considers a manufacturer-led Stackelberg game model within a closed-loop supply chain consisting of a dominant manufacturer (denoted as M), who acts as the leader, and a retailer (denoted as R) along with a third-party recycler (denoted as T) responsible for recycling, both acting as followers. The supply chain structure diagram is illustrated in Fig 1 below.

Given that w(t) is the wholesale price at time t when the manufacturer sets the new product (this paper assumes that the brand new product is perfectly homogeneous with the remanufactured product), it is a control variable for the manufacturer; p(t) is the retail price at time t when the retailer sets the new product, it is a control variable for the retailer; $c_{m}>0$ is the marginal production cost of the new product; $c_{r}>0$ is the marginal production cost of the remanufactured product; $\Delta =c_{m}-c_{r}>0$ is the cost saved on the remanufactured product, Δ>0 means that the cost of remanufactured products is lower than that of new products; r(t) is the recycling effort of the third-party recycler at time t, which is a control variable for the recycler, According to references [9,14], we employ a quadratic cost function for the recycling effort to capture the concept of diminishing returns to recycling investment. The function is represented as $\frac{\text{1}}{\text{2}}k_{T}r^{\text{2}}\left(t\right)$, and $k_{T}>0$; Assuming that the cumulative recycling cost back paid by the manufacturer to the recycler at time t is proportional to the recycling rate τ(t), that is γτ(t), γ>0 denotes the total payment made by the manufacturer when the recycling rate per unit of time is 1, then the recycling cost per unit of time is $\int_{\text{0}}^{\text{1}}\gamma \tau \left(t\right)dt$; T is the total length of time that the new product has been in the market; and N is the total number of potential consumers in the market.

In fact, the instantaneous product recovery rate exhibits a “cumulative effect,” meaning the recovery rate in a period is a function of past cumulative recovery efforts. According to the reference [13], the recycling rate is set as a state variable, and its rate of change is a function of the recycling effort and the recycling rate, which satisfies \stackrel·τ(t)=αr(t)−βτ(t), τ(0)=0. Where α is the coefficient of the recycling effort on the recycling rate, and β is the decline coefficient of the recycling rate, α,β>0. The key notations in this paper are summarized in Table 2 for ease of reference.

**Table 2:** Summary of notations.

Notation	Description
w(t)	Wholesale price at time t
p(t)	Retail price at time t
r(t)	Recycling effort of the third-party recycler at time t
$c_{m}$	Marginal production cost of the new product
$c_{r}$	Marginal production cost of the remanufactured product
$\Delta =c_{m}-c_{r}$	Cost saved on the remanufactured product
$k_{T}$	Recycling cost coefficient
$k_{T}r^{\text{2}}(t)/\text{2}$	Cost of the recycler’s recycling effort
τ(t)	Recycling rate
γ	Total payment made by the manufacturer when the recycling rate per unit of time is 1
$\int_{\text{0}}^{\text{1}}\gamma \tau \left(t\right)dt$	Recycling cost per unit of time *t*
T	Total length of time that the new product has been in the market
N	Total number of potential consumers in the market
α	Coefficient of the recycling effort on the recycling rate
β	Decline coefficient of the recycling rate
$\zeta_{\text{1}}$	Coefficient of purchase regret
$\zeta_{\text{2}}$	Coefficient of replacement regret
x(t)	Proportion of Innovators at time *t*
j	Consumer preference coefficient
k	Consumer imitation coefficient
D(t)	Innovative products’ demand at time *t*
a	Consumer’s price sensitivity coefficient

Assuming that consumers will have two kinds of regrets, purchase regret and replacement regret, and assuming that the number of consumer population with purchase regret is $\zeta_{\text{1}}x\left(t\right),0\leq \zeta_{\text{1}}\leq 1$. Purchase regret refers to the consumers who have purchased the product and regret the product, and the consumers who have purchase regret will not recommend the product to other consumers, which leads to slower diffusion of the product, then the proportion of innovators at time t satisfies $\dot{x}\left(t\right)=e^{-ap\left(t\right)}\left[j+k\left(1-\zeta_{\text{1}}\right)x\left(t\right)\right]\left(1-x\left(t\right)\right)$ when considering purchase regret; replacement regret refers to the consumers who have purchased the competing products and regret not purchasing the innovative product, which will become potential consumers of the innovative product in the Bass model, thus it will increase the number of imitators in the Bass model. Assuming that replacement regret increases the population of consumers as $\zeta_{\text{2}}x\left(t\right)$, $0\leq \zeta_{\text{2}}\leq 1$,then the proportion of innovators at time t when replacement regret is considered satisfies $\dot{x}\left(t\right)=e^{-ap\left(t\right)}\left[j+kx\left(t\right)\right]\left(1-x(t)+\zeta_{\text{2}}x\left(t\right)\right)=e^{-ap\left(t\right)}\left[j+kx\left(t\right)\right]\left(1-\left(1-\zeta_{\text{2}}\right)x\left(t\right)\right)$, then the proportion of innovators at time t when both regrets are considered is $\dot{x}\left(t\right)=e^{-ap\left(t\right)}\left[j+k\left(1-\zeta_{\text{1}}\right)x\left(t\right)\right]\left(1-\left(1-\zeta_{\text{2}}\right)x\left(t\right)\right)$; thus obtain the demand $D\left(t\right)=Ne^{-ap\left(t\right)}\left[j+k\left(1-\zeta_{\text{1}}\right)x\left(t\right)\right]\left(1-\left(1-\zeta_{\text{2}}\right)x\left(t\right)\right)$ for the innovative products at time t.

## 4. Modelling and solving

In this closed-loop supply chain, the dominant manufacturer first determines its wholesale price w(t), followed by the retailer who determines the retail price p(t), and the recycler who determines the recycling effort r(t). The long-run profit functions of the manufacturer, the retailer and the recycler are, respectively:


$$\begin{array}{c} \begin{array}{c} \pi_{M}=\overset{T}{\underset{\text{0}}{\int }}\left\{\left[w\left(t\right)-\left(1-\tau \left(t\right)\right)c_{m}-\tau \left(t\right)c_{r}\right]N\dot{x}\left(t\right)-\gamma \tau \left(t\right)\right\}dt\mathrm{\backslash hfill}\\ =\overset{T}{\underset{\text{0}}{\int }}\left\{\left[w\left(t\right)-c_{m}+\Delta \tau \left(t\right)\right]N\dot{x}\left(t\right)-\gamma \tau \left(t\right)\right\}dt\mathrm{\backslash hfill} \end{array} \end{array}$$ (5)

$$\begin{array}{c} \pi_{R}=\overset{T}{\underset{\text{0}}{\int }}\left[p\left(t)-w(t\right)\right]N\dot{x}\left(t\right)dt \end{array}$$ (6)

$$\begin{array}{c} \pi_{T}=\overset{T}{\underset{\text{0}}{\int }}\left[\gamma \tau \left(t)-\frac{\text{1}}{\text{2}}k_{T}r^{\text{2}}(t\right)\right]dt \end{array}$$ (7)

Further, manufacturers, retailers and recyclers each have their own objective functions:


$$\begin{array}{c} \underset{w\left(t\right)}{max}\left\{J_{M}=\overset{T}{\underset{\text{0}}{\int }}\left\{\left[w\left(t\right)-c_{m}+\Delta \tau \left(t\right)\right]N\dot{x}\left(t)-\gamma \tau (t\right)\right\}dt\right\} \end{array}$$ (8)

$$\begin{array}{c} \underset{p\left(t\right)}{max}\left\{J_{R}=\overset{T}{\underset{\text{0}}{\int }}\left[p\left(t)-w(t\right)\right]N\dot{x}\left(t\right)dt\right\} \end{array}$$ (9)

$$\begin{array}{c} \underset{r\left(t\right)}{max}\left\{J_{T}=\overset{T}{\underset{\text{0}}{\int }}\left[\gamma \tau \left(t)-\frac{\text{1}}{\text{2}}k_{T}r^{\text{2}}(t\right)\right]dt\right\} \end{array}$$ (10)

$$\begin{array}{c} s.t.\left\{ \begin{array}{c} @l@\dot{x}\left(t\right)=e^{-ap\left(t\right)}\left(j+k\left(1-\zeta_{\text{1}}\right)x\left(t\right)\right)\left(1-\left(1-\zeta_{\text{2}}\right)x\left(t\right)\right)\\ \dot{\tau }\left(t\right)=\alpha r\left(t\right)-\beta \tau \left(t\right)\\ x\left(0\right)=0\\ \tau \left(0\right)=0 \end{array}\right. \end{array}$$ (11)

**Proposition 1:** The manufacturer’s optimal wholesale price $w^{*}\left(t\right)$ is:


$$\begin{array}{c} \begin{array}{c} w^{*}\left(t\right)=\frac{\text{1}}{a}+c_{m}-\Delta \tau^{*}\left(t\right)\mathrm{\backslash hfill}\\ =\frac{\text{1}}{a}+c_{m}-\Delta \frac{\alpha^{\text{2}}\gamma }{k_{T}\beta^{\text{2}}}(1-\beta e^{\beta (T-t)}t-e^{-\beta t})\mathrm{\backslash hfill} \end{array} \end{array}$$ (12)

The retailer’s optimal wholesale price $p^{*}\left(t\right)$ is:


$$\begin{array}{c} \begin{array}{c} p^{*}\left(t\right)=\frac{\text{2}}{a}+c_{m}-\Delta \frac{\alpha^{\text{2}}\gamma }{k_{T}\beta^{\text{2}}}(1-\beta e^{\beta (T-t)}t-e^{-\beta t})\mathrm{\backslash hfill}\mathrm{\backslash vspace}1.5mm\\ \;\;\;-\frac{\text{1}}{a}ln\frac{\left(j+k\left(1-\zeta_{\text{1}}\right)x\left(T\right)\right)\left(1-\left(1-\zeta_{\text{2}}\right)x\left(T\right)\right)}{\left(j+k\left(1-\zeta_{\text{1}}\right)x\left(t\right)\right)\left(1-\left(1-\zeta_{\text{2}}\right)x\left(t\right)\right)}\mathrm{\backslash hfill} \end{array} \end{array}$$ (13)

The recycler’s optimal recycling effort $r^{*}\left(t\right)$ is:


$$\begin{array}{c} r^{*}\left(t\right)=\frac{\alpha \gamma }{k_{T}\beta }(1-e^{\beta (t-T)}) \end{array}$$ (14)

**Proof:** From the game sequence, the optimal recycling effort of the recycler and the optimal retail price of the retailer are first calculated.

The Hamilton function of the recycler is:


$$\begin{array}{c} H_{T}=\gamma \tau \left(t\right)-\frac{\text{1}}{\text{2}}k_{T}r^{\text{2}}\left(t\right)+\lambda_{T}\left(\alpha r\left(t)-\beta \tau (t\right)\right) \end{array}$$ (15)

From $\frac{\partial H_{T}}{\partial r\left(t\right)}=-k_{T}r\left(t\right)+\alpha \lambda_{T}\left(t\right)=0$, we can derive $r^{*}\left(t\right)=\frac{\alpha }{k_{T}}\lambda_{T}\left(t\right)$.

Given $\mathrm{\backslash stackrel}\cdot \lambda_{T}\left(t\right)=-\frac{\partial H_{T}}{\partial \tau \left(t\right)}=-\gamma +\beta \lambda_{T}\left(t\right)$, and the transversality condition $\lambda_{T}\left(T\right)=0$, it follows that


$$\begin{array}{c} \lambda_{T}\left(t\right)=\frac{\gamma }{\beta }(1-e^{\beta (t-T)}) \end{array}$$ (16)

So


$$\begin{array}{c} r^{*}\left(t\right)=\frac{\alpha \gamma }{k_{T}\beta }(1-e^{\beta (t-T)}) \end{array}$$ (17)

The Hamilton function for the retailer is:


$$\begin{array}{c} H_{R}=\left(Np\left(t\right)-Nw(t)+\lambda_{R}\left(t\right)\right)e^{-ap\left(t\right)}\left(j+k\left(1-\zeta_{\text{1}}\right)x\left(t\right)\right)\left(1-\left(1-\zeta_{\text{2}}\right)x\left(t\right)\right) \end{array}$$ (18)

From $\frac{\partial H_{R}}{\partial p}=0$, we can derive $p^{*}\left(t\right)$ as follows:


$$\begin{array}{c} p^{*}\left(t\right)=\frac{\text{1}}{a}+w\left(t\right)-\frac{\lambda_{R}\left(t\right)}{N} \end{array}$$ (19)

From


$$\begin{array}{c} \begin{array}{c} {\dot{\lambda }}_{R}\left(t\right)=-\frac{\partial H_{R}}{\partial x}=\frac{N}{a}e^{-ap\left(t\right)}\left(k\left(1-\zeta_{\text{1}}\right)-j(1-\zeta_{\text{2}})-2k\left(1-\zeta_{\text{1}}\right)\left(1-\zeta_{\text{2}}\right)x\left(t\right)\right)\mathrm{\backslash vspace}1.5mm\mathrm{\backslash hfill}\\ \;\;\;=-\dot{x}\left(t\right)\frac{N\left(k\left(1-\zeta_{\text{1}}\right)-j(1-\zeta_{\text{2}})-2k\left(1-\zeta_{\text{1}}\right)\left(1-\zeta_{\text{2}}\right)x\left(t\right)\right)}{a\left(j+k\left(1-\zeta_{\text{1}}\right)x\left(t\right)\right)\left(1-\left(1-\zeta_{\text{2}}\right)x\left(t\right)\right)}\mathrm{\backslash hfill} \end{array} \end{array}$$ (20)

and the transversality condition $\lambda_{R}\left(T\right)=0$, we can derive:


$$\begin{array}{c} \lambda_{R}\left(t\right)=\frac{N}{a}ln\frac{\left(j+k\left(1-\zeta_{\text{1}}\right)x\left(T\right)\right)\left(1-\left(1-\zeta_{\text{2}}\right)x\left(T\right)\right)}{\left(j+k\left(1-\zeta_{\text{1}}\right)x\left(t\right)\right)\left(1-\left(1-\zeta_{\text{2}}\right)x\left(t\right)\right)} \end{array}$$ (21)

$$\begin{array}{c} p^{*}\left(t\right)=\frac{\text{1}}{a}+w^{*}\left(t\right)-\frac{\text{1}}{a}ln\frac{\left(j+k\left(1-\zeta_{\text{1}}\right)x\left(T\right)\right)\left(1-\left(1-\zeta_{\text{2}}\right)x\left(T\right)\right)}{\left(j+k\left(1-\zeta_{\text{1}}\right)x\left(t\right)\right)\left(1-\left(1-\zeta_{\text{2}}\right)x\left(t\right)\right)} \end{array}$$ (22)

Thus $\mathrm{\backslash stackrel}\cdot x\left(t\right)=e^{-1-aw\left(t\right)}\left(j+k\left(1-\zeta_{\text{1}}\right)x\left(T\right)\right)\left(1-\left(1-\zeta_{\text{2}}\right)x\left(T\right)\right)$.

Finally the optimal wholesale price is calculated and the manufacturer’s Hamilton function is:


$$\begin{array}{c} \begin{array}{c} H_{M}=\left(Nw\left(t\right)-Nc_{m}+N\Delta \tau (t)+\lambda_{M}\left(t\right)\right)e^{-1-aw\left(t\right)}\left(j+k\left(1-\zeta_{\text{1}}\right)x\left(T\right)\right)\left(1-\left(1-\zeta_{\text{2}}\right)x\left(T\right)\right)\mathrm{\backslash hfill}\\ \;\;-\gamma \tau \left(t\right)\mathrm{\backslash hfill} \end{array} \end{array}$$ (23)

From


$$\begin{array}{c} \begin{array}{c} \frac{\partial H_{M}}{\partial w}=(N-aNw(t)+aNc_{m}-aN\Delta \tau (t)\mathrm{\backslash hfill}\\ \;\;-a\lambda_{M}(t))e^{-\left(1+aw\left(t\right)\right)}\left(j+k(1-\zeta_{\text{1}})x\left(T\right)\right)\left(1-(1-\zeta_{\text{2}})x\left(T\right)\right)\mathrm{\backslash hfill}\\ \;\;=0\mathrm{\backslash hfill} \end{array} \end{array}$$ (24)

$$\begin{array}{c} we\;can\;derive\text{\textbackslash{}hspace\{0.17em}\}w^{*}\left(t\right)=\frac{\text{1}}{a}+c_{m}-\Delta \tau \left(t\right)-\frac{\lambda_{M}\left(t\right)}{N} \end{array}$$ (25)

From $\mathrm{\backslash stackrel}\cdot \lambda_{M}\left(t\right)=-\frac{\partial H_{M}}{\partial x}=0$ and the transversality condition $\lambda_{M}\left(T\right)=0$ we can derive $\lambda_{M}\left(t\right)=0$.

From \stackrel·τ(t)=αr(t)−βτ(t), $r^{*}\left(t\right)=\frac{\alpha \gamma }{k_{T}\beta }(1-e^{\beta (t-T)})$ and τ(0)=0 we can derive:


$$\begin{array}{c} \tau^{*}\left(t\right)=\frac{\alpha^{\text{2}}\gamma }{k_{T}\beta^{\text{2}}}\left(1-e^{-\beta t}+\frac{\text{1}}{\text{2}}e^{-\beta T}\left(e^{-\beta t}-e^{\beta t}\right)\right) \end{array}$$ (26)

So


$$\begin{array}{c} w^{*}\left(t\right)=\frac{\text{1}}{a}+c_{m}-\Delta \frac{\alpha^{\text{2}}\gamma }{k_{T}\beta^{\text{2}}}\left(1-e^{-\beta t}+\frac{\text{1}}{\text{2}}e^{-\beta T}\left(e^{-\beta t}-e^{\beta t}\right)\right) \end{array}$$ (27)

$$\begin{array}{c} \begin{array}{c} p^{*}\left(t\right)=\frac{\text{2}}{a}+c_{m}-\Delta \frac{\alpha^{\text{2}}\gamma }{k_{T}\beta^{\text{2}}}\left(1-e^{-\beta t}+\frac{\text{1}}{\text{2}}e^{-\beta T}\left(e^{-\beta t}-e^{\beta t}\right)\right).\mathrm{\backslash vspace}1.5mm\mathrm{\backslash hfill}\\ \;\;-\frac{\text{1}}{a}ln\frac{\left(j+k\left(1-\zeta_{\text{1}}\right)x\left(T\right)\right)\left(1-\left(1-\zeta_{\text{2}}\right)x\left(T\right)\right)}{\left(j+k\left(1-\zeta_{\text{1}}\right)x\left(t\right)\right)\left(1-\left(1-\zeta_{\text{2}}\right)x\left(t\right)\right)}\mathrm{\backslash hfill} \end{array} \end{array}$$ (28)

**Proposition 2:** The optimal innovator proportion path $x^{*}\left(t\right)$ for this innovative products is:


$$\begin{array}{c} x^{*}\left(t\right)=\left(j+k\left(1-\zeta_{\text{1}}\right)x\left(T\right)\right)\left(1-\left(1-\zeta_{\text{2}}\right)x\left(T\right)\right)B\left(t\right) \end{array}$$ (29)

Where $B\left(t\right)=\int_{\text{0}}^{t}e^{-2-ac_{m}+a\Delta \frac{\alpha^{\text{2}}\gamma }{k_{T}\beta^{\text{2}}}\left(1-e^{-\beta t}+\frac{\text{1}}{\text{2}}e^{-\beta T}\left(e^{-\beta t}-e^{\beta t}\right)\right)}dt$, $x\left(T\right)=\frac{-1-B\left(T\right)j(1-\zeta_{\text{2}})+B\left(T\right)k(1-\zeta_{\text{1}})+\sqrt{4B^{\text{2}}\left(T\right)jk(1-\zeta_{\text{1}})(1-\zeta_{\text{2}})+(-1-B\left(T\right)j\left(1-\zeta_{\text{2}}\right)+B\left(T\right)k\left(1-\zeta_{\text{1}}\right){)}^{\text{2}}}}{2B\left(T\right)k(1-\zeta_{\text{1}})(1-\zeta_{\text{2}})}$.

The optimal recycling rate path $\tau^{*}\left(t\right)$ is:


$$\begin{array}{c} \tau^{*}\left(t\right)=\frac{\alpha^{\text{2}}\gamma }{\beta^{\text{2}}k_{T}}\left(1-e^{-\beta t}+\frac{\text{1}}{\text{2}}e^{-\beta T}\left(e^{-\beta t}-e^{\beta t}\right)\right) \end{array}$$ (30)

**Proof:** The proof of the path $\tau^{*}\left(t\right)$ of the optimal recycling rate is given in the proof of Proposition 1.

From Proposition 1


$$\begin{array}{c} \mathrm{\backslash stackrel}\cdot x\left(t\right)=e^{-2-ac_{m}+a\Delta \frac{\alpha^{\text{2}}\gamma }{k_{T}\beta^{\text{2}}}\left(1-e^{-\beta t}+\frac{\text{1}}{\text{2}}e^{-\beta T}\left(e^{-\beta t}-e^{\beta t}\right)\right)}\left(j+k\left(1-\zeta_{\text{1}}\right)x\left(T\right)\right)\left(1-\left(1-\zeta_{\text{2}}\right)x\left(T\right)\right) \end{array}$$ (31)

And x(0)=0, we can derive:


$$\begin{array}{c} \begin{array}{c} x\left(t\right)=\left(j+k\left(1-\zeta_{\text{1}}\right)x\left(T\right)\right)(1-(1-\\ \;\zeta_{\text{2}})x\left(T\right))\int_{\text{0}}^{t}e^{-2-ac_{m}+a\Delta \frac{\alpha^{\text{2}}\gamma }{k_{T}\beta^{\text{2}}}\left(1-e^{-\beta t}+\frac{\text{1}}{\text{2}}e^{-\beta T}\left(e^{-\beta t}-e^{\beta t}\right)\right)}dt \end{array} \end{array}$$ (32)

Let $B\left(t\right)=\int_{\text{0}}^{t}e^{-2-ac_{m}+a\Delta \frac{\alpha^{\text{2}}\gamma }{k_{T}\beta^{\text{2}}}\left(1-e^{-\beta t}+\frac{\text{1}}{\text{2}}e^{-\beta T}\left(e^{-\beta t}-e^{\beta t}\right)\right)}dt$, Thus


$$\begin{array}{c} x\left(t\right)=\left(j+k\left(1-\zeta_{\text{1}}\right)x\left(T\right)\right)\left(1-\left(1-\zeta_{\text{2}}\right)x\left(T\right)\right)B\left(t\right) \end{array}$$ (33)

When t=T, $x\left(T\right)=\left(j+k\left(1-\zeta_{\text{1}}\right)x\left(T\right)\right)\left(1-\left(1-\zeta_{\text{2}}\right)x\left(T\right)\right)B\left(T\right)$

we can obtain:


$$\begin{array}{c} x\left(T\right)=\frac{-1-B\left(T\right)j(1-\zeta_{\text{2}})+B\left(T\right)k(1-\zeta_{\text{1}})+\sqrt{4B^{\text{2}}\left(T\right)jk(1-\zeta_{\text{1}})(1-\zeta_{\text{2}})+(-1-B\left(T\right)j\left(1-\zeta_{\text{2}}\right)+B\left(T\right)k\left(1-\zeta_{\text{1}}\right){)}^{\text{2}}}}{2B\left(T\right)k(1-\zeta_{\text{1}})(1-\zeta_{\text{2}})} \end{array}$$ (34)

Or


$$\begin{array}{c} x\left(T\right)=\frac{-1-B\left(T\right)j(1-\zeta_{\text{2}})+B\left(T\right)k(1-\zeta_{\text{1}})-\sqrt{4B^{\text{2}}\left(T\right)jk(1-\zeta_{\text{1}})(1-\zeta_{\text{2}})+(-1-B\left(T\right)j\left(1-\zeta_{\text{2}}\right)+B\left(T\right)k\left(1-\zeta_{\text{1}}\right){)}^{\text{2}}}}{2B\left(T\right)k(1-\zeta_{\text{1}})(1-\zeta_{\text{2}})}<0 \end{array}$$ (35)

By substituting the variables into equations (5–7), the optimal long-term profits for the manufacturer, retailer, and recycler are as follows:


$$\begin{array}{c} \pi_{M}=\frac{N}{a}x\left(T\right)-\frac{\alpha^{\text{2}}\gamma^{\text{2}}}{2k_{T}\beta^{\text{3}}}(2\beta T+4e^{-\beta T}-e^{-2\beta T}-3) \end{array}$$ (36)

$$\begin{array}{c} \pi_{R}=\frac{N}{a}x\left(T\right)-\int_{\text{0}}^{x\left(T\right)}\frac{N}{a}ln\frac{\left(j+k\left(1-\zeta_{\text{1}}\right)x\left(T\right)\right)\left(1-\left(1-\zeta_{\text{2}}\right)x\left(T\right)\right)}{\left(j+k\left(1-\zeta_{\text{1}}\right)x\left(t\right)\right)\left(1-\left(1-\zeta_{\text{2}}\right)x\left(t\right)\right)}dx\left(t\right) \end{array}$$ (37)

$$\begin{array}{c} \pi_{T}=\frac{\alpha^{\text{2}}\gamma^{\text{2}}}{4k_{T}\beta^{\text{3}}}(2\beta T+4e^{-\beta T}-e^{-2\beta T}-3) \end{array}$$ (38)

**Result 1:** From Eq. (12), it can be seen that the manufacturer’s optimal wholesale price is not fixed, but from equation $w^{*}\left(t\right)-c_{m}-(1-\Delta )\tau \left(t\right)=\frac{\text{1}}{a}$, we can see that the manufacturer’s marginal profit per new product is fixed. And from equation (12), it can be seen that the manufacturer’s optimal wholesale price does not contain $\zeta_{\text{1}}$, $\zeta_{\text{2}}$, that is, the manufacturer’s optimal wholesale price is not affected by the consumer purchase regret.

**Result 2:** From Eq. (13), it can be seen that retailers, unlike manufacturers, also have marginal margins that vary in real time as $p^{*}\left(t\right)-w^{*}\left(t\right)=\frac{\text{1}}{a}-\frac{\text{1}}{a}ln\frac{\left(j+k\left(1-\zeta_{\text{1}}\right)x\left(T\right)\right)\left(1-\left(1-\zeta_{\text{2}}\right)x\left(T\right)\right)}{\left(j+k\left(1-\zeta_{\text{1}}\right)x\left(t\right)\right)\left(1-\left(1-\zeta_{\text{2}}\right)x\left(t\right)\right)}$; it can be seen that consumers’ purchase regret and replacement regret behaviors will affect the retailer’s optimal retail price, and $\frac{\partial p^{*}\left(t\right)}{\partial \zeta_{\text{1}}}=\frac{\text{1}}{a}\frac{jk\left(x\left(T\right)-x\left(t\right)\right)}{\left(j+kx\left(T\right)\left(1-\zeta_{\text{1}}\right)\right)\left(j+kx\left(t\right)\left(1-\zeta_{\text{1}}\right)\right)}$, since x(t) is the proportion of innovators at time t, and is monotonically increasing with t, therefore x(T)−x(t)≥0, and only when t=T, x(T)−x(t)=0. In addition, because j,k>0,x(t)≥0, $0\leq \zeta_{\text{1}},\zeta_{\text{2}}\leq 1$, then $\left(j+kx\left(T\right)\left(1-\zeta_{\text{1}}\right)\right)\left(j+kx\left(t\right)\left(1-\zeta_{\text{1}}\right)\right)>0$. Therefore, $\frac{\partial p^{*}\left(t\right)}{\partial \zeta_{\text{1}}}=\frac{\text{1}}{a}\frac{jk\left(x\left(T\right)-x\left(t\right)\right)}{\left(j+kx\left(T\right)\left(1-\zeta_{\text{1}}\right)\right)\left(j+kx\left(t\right)\left(1-\zeta_{\text{1}}\right)\right)}\geq 0$.If and only if t=T, $\frac{\partial p^{*}\left(t\right)}{\partial \zeta_{\text{1}}}=0$, that is, the larger the purchase regret coefficient $\zeta_{\text{1}}$ is will increase. Similarly, $\frac{\partial p^{*}\left(t\right)}{\partial \zeta_{\text{2}}}=\frac{\text{1}}{a}\frac{x\left(t\right)-x\left(T\right)}{\left(1-x\left(T\right)\left(1-\zeta_{\text{2}}\right)\right)\left(1-x\left(t\right)\left(1-\zeta_{\text{2}}\right)\right)}\leq 0$, that is, the larger the replacement regret coefficient $\zeta_{\text{2}}$ is, the prices at all times except t=T will decrease.

**Result 3:** From Eq. (14), it can be seen that the recycler’s optimal recycling effort also changes in real time. Since the expression of $r^{*}\left(t\right)$ does not contain $\zeta_{\text{1}},\zeta_{\text{2}}$, the recycler’s optimal recycling effort is not affected by consumers’ post-purchase regret. And $\frac{dr^{*}\left(t\right)}{dt}=-\frac{\alpha \gamma }{k_{T}}e^{\beta (t-T)}<0$, indicating that the recycler’s optimal recycling effort decreases over time until T, $r^{*}\left(T\right)=\frac{\alpha \gamma }{k_{T}\beta }(1-e^{\beta (T-T)})=0$. Similarly, from Eq. (26), it can be seen that the change path of the recycling rate is also not affected by consumers’ post-purchase regret.

## 5. Analysis of the calculations

Due to the complexity of the function, the impacts of the key parameters on profits and other variables in the paper cannot be determined directly by analytical methods. Therefore, this section will illustrate the impact of each parameter on the profit of members on closed-loop supply chains through numerical simulation.

### 5.1. Impact of purchase regret and replacement regret on sales and profits

According to reference [33], We assume that other parameters on this closed-loop supply chains are constant as shown in Table 3. Then the impact of the purchase regret coefficient $\zeta_{\text{1}}$ and the replacement regret coefficient $\zeta_{\text{2}}$ on the total final sales volume and the long-term profit of each member of the supply chain is shown below.

**Table 3:** Closed-loop Supply Chains Parameter Assignments (1).

a	$c_{m}$	$c_{r}$	Δ	N
0.004	100	50	50	100000
$k_{T}$	α	β	γ	T
20000	0.2	0.5	100000	30

**Fig 2:**
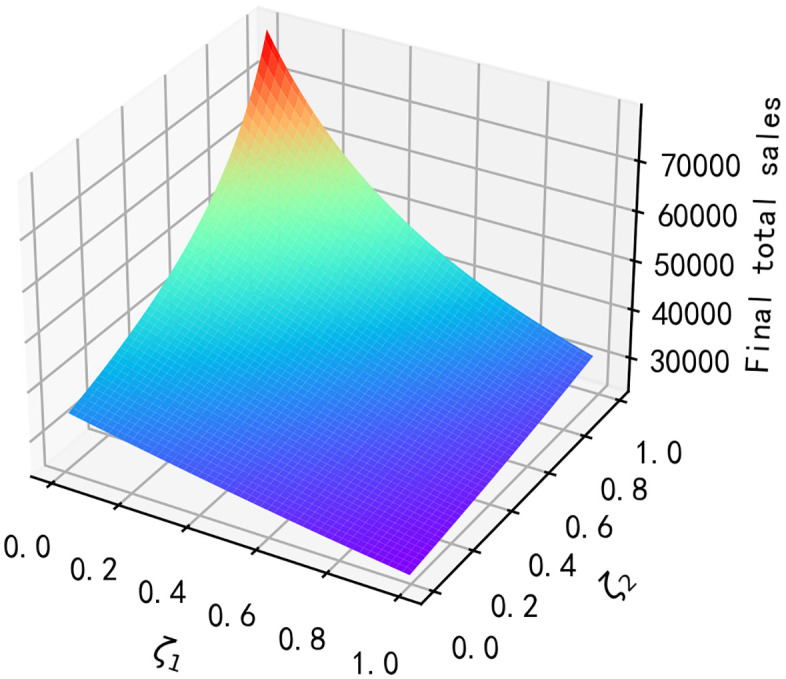
Impact of $\zeta_{1}$, $\zeta_{2}$ on total final sales.

**Fig 3:**
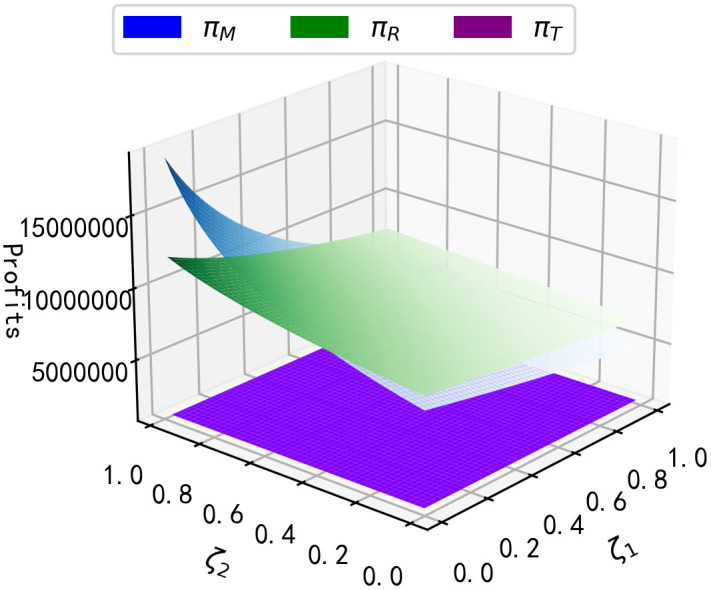
Impact of $\zeta_{1}$, $\zeta_{2}$ on long-term profits.

**Result 4:** As seen in Fig 2, total final sales of the product decrease with increasing purchase regret coefficients $\zeta_{\text{1}}$ and increase with increasing replacement regret coefficients $\zeta_{\text{2}}$; as seen in Fig 3, manufacturer’s long-run profits and retailer’s long-run profits decrease with increasing purchase regret coefficients $\zeta_{\text{1}}$; however, as replacement regret coefficients $\zeta_{\text{2}}$ increase with increasing replacement regret coefficients, so do the long-run profits of the manufacturer and retailer; and the long-run profits of the third-party recycler are unaffected by the impact of consumer regret behaviour. It can be seen that as the replacement regret coefficient $\zeta_{\text{2}}$ increases, manufacturers increase their profits more than retailers, and as $\zeta_{\text{2}}$ increases, manufacturers’ long-term profits will be higher than retailers’ long-term profits.

### 5.2. Impact of product to market time length T and purchase regret on profits

**Fig 4:**
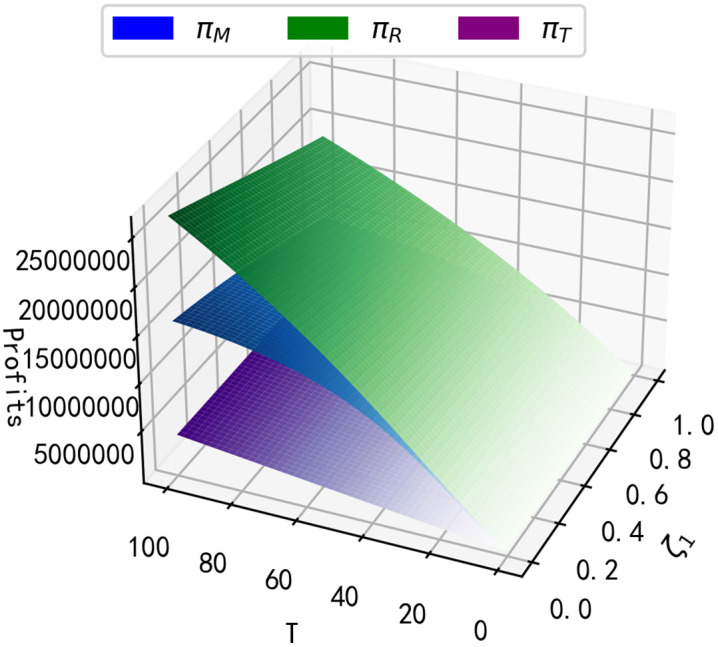
Impact of T and $\zeta_{1}$ on long-term profits.

According to reference [33], We assume that the other parameters are as shown in Table 4, the impact of T and $\zeta_{\text{1}}$ on the long term profit of each member of the supply chain is obtained by varying the values of T and $\zeta_{\text{1}}$ as shown in Fig 4.

**Table 4:** Closed-loop Supply Chains Parameter Assignment (2).

a	$c_{m}$	$c_{r}$	Δ	N
0.004	100	50	50	100000
$k_{T}$	α	β	γ	$\zeta_{\text{2}}$
20000	0.2	0.5	100000	0.1

**Fig 5:**
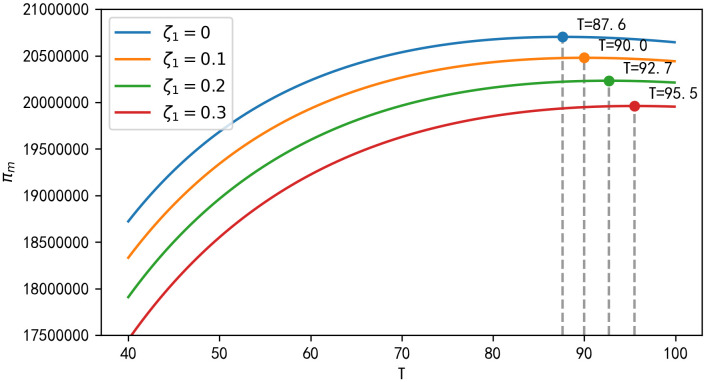
Impact of T and $\zeta_{1}$ on manufacturers’ long-term profits.

**Result 5:** As seen in Fig 4, the long-run profits of the manufacturer and the retailer decrease as the purchase regret coefficient $\zeta_{\text{1}}$ increases, and the long-run profits of the retailer and the third-party recycler increase as the length of time T the product is on the market continues to increase. As seen in Fig 5, there exists one $\hat{T}$ for the manufacturer that maximises the manufacturer’s long-run profit, and the manufacturer’s optimal length of time $\hat{T}$ on the market lengthens as the purchase regret coefficient $\zeta_{\text{1}}$ increases. For example, when $\zeta_{\text{1}}=0$, the optimal time length $\hat{T}=87.6$; at $\zeta_{\text{1}}=0.1$, $\hat{T}$ increases to 90 (a rise of 2.4 compared to $\zeta_{\text{1}}=0$); and at $\zeta_{\text{1}}=0.2$, $\hat{T}$ further increases to 92.7, representing increments of 5.1 and 2.7 relative to $\zeta_{\text{1}}=0$ and $\zeta_{\text{1}}=0.1$, respectively.

### 5.3. Impact of product to market time length T and replacement regret on profits

**Fig 6:**
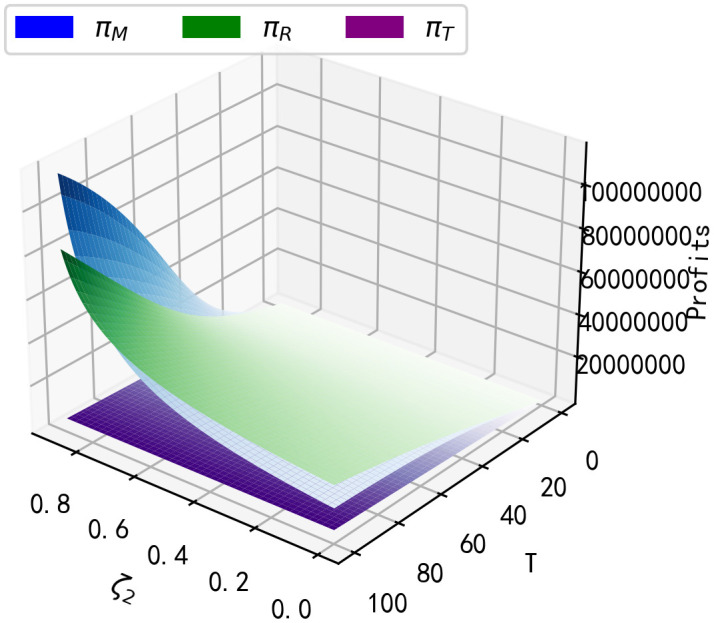
Impact of T and $\zeta_{2}$ on long-term profits.

According to reference [33], We assume that the other parameters are as shown in Table 5, the impact of T and $\zeta_{\text{2}}$ on the long term profit of each member of the supply chain is obtained by changing the values of T and $\zeta_{\text{2}}$ as shown in Fig 6.

**Table 5:** Closed-loop Supply Chains Parameter Assignments (3).

a	$c_{m}$	$c_{r}$	Δ	N
0.004	100	50	50	100000
$k_{T}$	α	β	γ	$\zeta_{\text{1}}$
20000	0.2	0.5	100000	0.1

**Fig 7:**
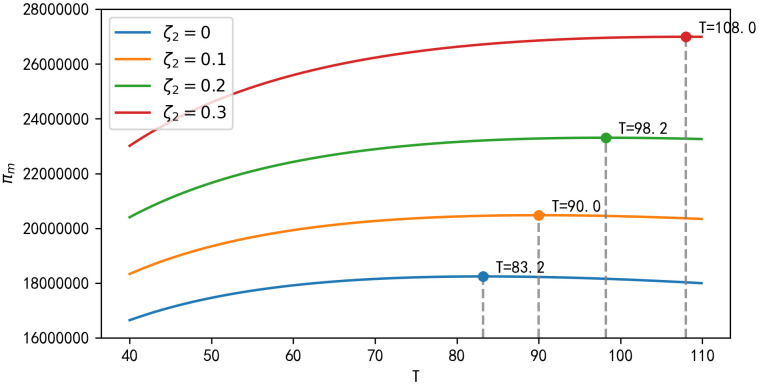
Impact of T and $\zeta_{2}$ on long-term profits of manufacturers.

**Result 6:** As can be seen in Fig 6, the long-run profits of the manufacturer and retailer increase with the replacement regret coefficient $\zeta_{\text{2}}$. Similar to Result 5, the long-run profits of the retailer and the third-party recycler increase as the product’s time-to-market length T continues to increase. And as can be seen in Fig 7, as the replacement regret coefficient $\zeta_{\text{2}}$ increases, the manufacturer’s optimal length of time on the market $\hat{T}$ also lengthens. For example, when $\zeta_{\text{2}}=0$, the optimal time length $\hat{T}=108$; at $\zeta_{\text{2}}=0.1$, $\hat{T}$ decreases to 98.2 (a decrease of 9.8 compared to $\zeta_{\text{2}}=0$); and at $\zeta_{\text{2}}=0.2$, $\hat{T}$ further declines to 90, representing decrements of 18 and 8.2 relative to $\zeta_{\text{2}}=0$ and $\zeta_{\text{2}}=0.1$, respectively.

### 5.4. The impact of dual regret on the manufacturer’s optimal market entry duration

**Fig 8:**
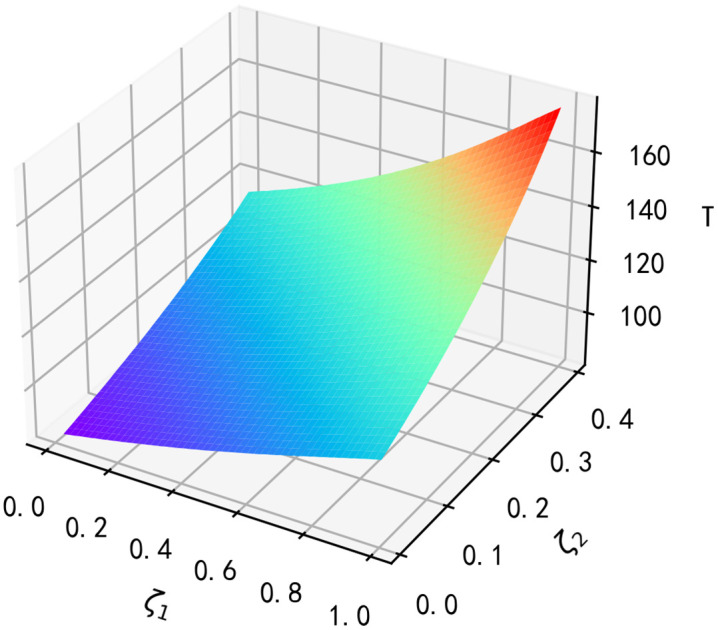
Impact of $\zeta_{1}$ and $\zeta_{2}$ on the manufacturer’s optimal length of time on the market.

From results 5 and 6, purchase regret and replacement regret will affect the manufacturer’s optimal length of time in the market, and assuming that the other parameters are as shown in Table 6, the impact of $\zeta_{\text{1}}$ and $\zeta_{\text{2}}$ on the manufacturer’s optimal length of time in the market is shown in Fig 8.

**Table 6:** Closed-loop Supply Chains Parameter Assignment (4).

a	$c_{m}$	$c_{r}$	Δ	N
0.004	100	50	50	100000
$k_{T}$	α	β	γ	
20000	0.2	0.5	100000	

**Result 7:** As seen in Fig 8, the manufacturer’s optimal length of time on the market T increases with the increase of the purchase regret coefficient $\zeta_{\text{1}}$ and the replacement regret coefficient $\zeta_{\text{2}}$; and the impact of the purchase regret coefficient $\zeta_{\text{1}}$ on the optimal length of time on the market is smaller than the impact of the replacement regret coefficient $\zeta_{\text{2}}$. For example, when $\zeta_{\text{1}}=0$, $\zeta_{\text{2}}=0$, $\zeta_{\text{1}}$ is lifted by 0.01, the optimal duration increases by 0.2, and $\zeta_{\text{2}}$ is lifted by 0.01, the optimal duration increases by 0.6; When $\zeta_{\text{1}}=0.1$, $\zeta_{\text{2}}=0.1$, $\zeta_{\text{1}}$ is lifted by 0.01, the optimal duration increases by 0.3, and $\zeta_{\text{2}}$ is lifted by 0.01, the optimal duration increases by 0.8.

When the purchase regret coefficient increases, the manufacturer’s optimal length of time on the market will increase, but the increase is relatively small. This is because the impact of purchase regret behavior on the product diffusion speed is relatively small. In contrast, the replacement regret coefficient has a greater impact on the optimal length of time on the market. This is because replacement regret behavior involves the existence of competing products, and consumers’ dissatisfaction with substitute products may prompt them to be more willing to purchase innovative products, thus prolonging the product’s market diffusion time.

## 6. Conclusions

This paper researches the dynamic closed-loop supply chains of innovative products market diffusion, in which the manufacturer leads, the retailer and the third-party recycler follow, and the consumer’s purchase regret and replacement regret are taken into account; Through the differential games model, the optimal wholesale price path of the manufacturer $w^{*}\left(t\right)$, the optimal retail price path of the retailer $p^{*}\left(t\right)$ and the optimal recycling effort path of the third-party recycler $r^{*}\left(t\right)$ are obtained to further maximise profits for each member; and the following conclusions are drawn from the analyses of the examples:

(1) From the result 1, the manufacturer’s optimal retail price strategy is: regardless of the recycling rate and market demand changes, always ensure that each innovative product to obtain the marginal profit of $\frac{\text{1}}{a}$, so as to enable it to obtain the maximum profit, and the manufacturer’s pricing strategy is not affected by the both types of consumer post-purchase regret, which indicates that in the appearance of the consumer post-purchase regret behaviour, the manufacturer still maintains the original wholesale pricing strategy; and from result 2, it can be seen that consumer purchase regret behaviour affects the retailer’s optimal retail price, and the more serious the consumer purchase regret behaviour is, the higher the optimal retail price is, which shows that when consumer purchase regret occurs frequently, retailers have to increase the retail price of innovative products to make up for the loss of profit due to consumer regret; while when consumer purchase regret occurs, retailers should lower the optimal retail price to attract more consumers in order to gain more profit. From result 3, the optimal recycling effort strategy of the recycler is to increase the recycling rate with higher recycling effort in the initial period, and then gradually reduce the recycling effort to maintain a higher employment recycling cost until the product is withdrawn from the market, then no more recycling will be done; and the recycling effort strategy is not affected by the both types of consumer post-purchase regret.
(2) From result 4, it can be seen that consumer purchase regret will lead to a decline in the sales of the innovative product, because consumer purchase regret behaviour affects the spread of the innovative product among consumers and slows down the diffusion of the product in the market, which leads to a decline in the final total sales of the product, and further leads to a decline in the manufacturer‘s long-term profit; the retailer can only make up for some of the lost profit caused by consumer purchase regret by increasing the retail price, but it cannot recoup all the losses caused by the consumer purchase regret, which leads to a decline in its long-term profit. Therefore, consumer purchase regret needs to be mitigated by providing better after-sales service to improve consumer satisfaction. The consumer replacement regret will lead to an increase in the sales of the innovative product due to the dissatisfaction of the consumers of the competing products, thus indicating that the innovative product is better than the competing product, which will influence the potential consumers of the better innovative product to buy the innovative product, resulting in higher total final sales of the product, and further higher long-term profits for the manufacturer and retailer.
(3) As shown in results 5 and 6, The duration T when products are introduced to the market varies, the maximum long-term profit of each member of the closed-loop supply chains is different depending on the length of the product‘s time on the market; for retailers and third-party recyclers, the longer the length of the product‘s time on the market, the larger the profit they will obtain, and for manufacturers, there exists an optimal length of the product‘s time on the market, which will decrease the manufacturer‘s long-term profit when the length is greater than or less than that.
(4) And from result 7, it can be seen that the creation of consumer purchase regret behaviour and replacement regret behaviour both lead to a longer time for the manufacturer to obtain optimal profit. This is because purchase regret leads to a decrease in the innovative population and affects the diffusion speed of innovative products, and in order to sell all the products produced, it will definitely take longer time for innovative products to diffuse in the market. The generation of replacement regret behaviour expands the scale of potential consumers, and under the premise that the diffusion speed of innovative products is fixed, to influence all potential consumers will certainly prolong the diffusion time of innovative products.

Furthermore, we obtain the following management implications:

(1) Manufacturer enterprises should ensure that their wholesale price policy can maintain the stability of marginal profits and are not affected by market fluctuations and consumers’ post-purchase regret behaviors. This can be achieved by formulating pricing policy, establishing stable supply chains, and implementing cost control measures.
(2) Retailer enterprises should be aware of the impact of consumers’ purchase regret behaviors on their optimal retail prices and adjust their pricing policy accordingly. When purchase regret is severe, they can moderately increase the retail price to make up for the lost profits. When replacement regret behaviors exist, they can lower the optimal retail price to attract more consumers.
(3) Manufacturer and retailer enterprises should be aware of the impact of consumers’ purchase regret behaviors and replacement regret behaviors on product sales volume and long-term profits. To mitigate the negative impact of purchase regret behaviors on product diffusion, they can consider providing better after-sales services to improve consumers’ satisfaction, thereby reducing the occurrence of purchase regret. And they can also strengthen product experiences and publicize the advantages of products to stimulate consumers to have replacement regret.
(4) Managers need to recognize the impact of consumers’ purchase regret behaviors and replacement regret behaviors on prolonging the product diffusion time. When formulating market plans and sales expectations, they should take the existence of these behaviors into account and adjust strategies and schedules accordingly.

In this paper, two consumer post-purchase regret are considered, and the innovative products closed-loop supply chains, in which third-party recyclers are responsible for recycling, are researched, and the remanufactured products are assumed to be homogeneous with the new products. The optimal decisions of each member of the closed-loop supply chains are obtained through the differential game method, and the effects of consumer purchase regret and replacement regret on the closed-loop supply chains are analysed. Subsequently study that remanufactured products and new products are heterogeneous closed-loop supply chains, or consider closed-loop supply chains differential games where products do not diffuse in a continuous manner, but in a discrete manner.

## Supporting information

S1 Data The minimal data set. (RAR)
